# Antimalarial Activity of Extract and Fractions of *Sauropus androgynus* (L.) Merr.

**DOI:** 10.1155/2022/3552491

**Published:** 2022-09-08

**Authors:** Wiwied Ekasari, Dwi Fatmawati, Siti M. Khoiriah, Wenda A. Baqiuddin, Hawi Q. Nisa, Adinda A. S. Maharupini, Tutik S. Wahyuni, Rice D. Oktarina, Eko Suhartono, Ram K. Sahu

**Affiliations:** ^1^Department of Pharmaceutical Sciences, Faculty of Pharmacy, Airlangga University, Surabaya 60115, East Java, Indonesia; ^2^Department of Medical Chemistry/Biochemistry, Faculty of Medicine, Lambung Mangkurat University, Banjarmasin 70122, South Kalimantan, Indonesia; ^3^Department of Pharmaceutical Science, Assam University (A Central University), Silchar 788011, Assam, India

## Abstract

*Sauropus androgynus* (L.) Merr., in the Indonesian local name known as “Katuk,” is a tropical shrub plant of the family Euphorbiaceae. Based on genus and chemotaxonomic approaches, as well as in vitro testing of *Plasmodium falciparum*, leaves of *S. androgynus* are presumed to have an active compound content as an antimalarial. The current study aims to investigate the antimalarial activity of 96% ethanol extract and fractions of *S. androgynus* leaves. The ethanolic extract was fractionated using the vacuum liquid chromatography (VLC) method with three solvents of different polarities (n-hexane, chloroform, and 96% ethanol). The fraction obtained was then evaluated for antimalarial activity against *P. falciparum* 3D7 strain. The ethanolic extract was evaluated for antimalarial suppressive and prophylactic activity against *P. berghei*-infected mice, as well as inhibitory activity against the heme detoxification process in vitro. Fractionation of ethanolic extract resulted in seven combined fractions, with the most active fraction being FV (50% inhibitory concentration (IC_50_) = 2.042 *µ*g/mL). The ethanolic extract showed good parasitic suppressive (therapeutic) activity with a median effective dose (ED_50_) value of 15.35 mg/kg body weight. In a prophylactic test, ethanolic extract showed parasite growth inhibitory activity of 67.74 ± 9.21% after the administration of 400 mg/kg body weight for 4 days before infection, and 65.30 ± 10.44% after the administration of 200 mg/kg body weight for 8 consecutive days (4 days before and after infection). The ethanolic extract also showed an effect in inhibiting the formation of *β*-hematin of about 26.87–79.36% at a concentration of 0.1–4 mg/mL and an IC_50_ value of 0.479 mg/mL. The *S. androgynus* leaves were shown to have antimalarial activity in vitro and in vivo, where ethanolic extract were more active compared with the fraction obtained. The antimalarial properties of the extract showed a higher suppressive activity than prophylactic activity.

## 1. Introduction

Malaria is an infectious disease caused by a protozoan parasite of the genus *Plasmodium*. The spread of *Plasmodium* into the human body is through the bite of female mosquitoes of the genus *Anopheles*. Malaria in humans is caused by five parasitic species of the genus *Plasmodium*, including *P. falciparum*, *P. vivax*, *P. ovale*, *P. malariae*, and *P. knowlesi* [1]. Malaria is one of the infectious diseases that can cause death, anemia, and decrease work productivity [2, 3].

Globally, there are an estimated 241 million malaria cases in 85 malaria-endemic countries in 2020, and there is a 12% increase in malaria-related deaths compared with 2019 [4]. Nationally, the number of malaria cases in Indonesia has decreased by more than 50% from 465,764 cases (annual parasite incidence (API) = 1.96%) in 2010 to 222,085 cases (API = 0.84%) in 2018 [5]. However, there was a stagnation in malaria cases after 2014, which indicates that interventions in elimination programs that have already been implemented are not enough to reduce cases drastically.

Efforts in controlling malaria are essential including prevention, diagnosis, and treatment. The diagnosis of malaria can be seen from the results of the examination of blood preparations, while chloroquine is used to treat malaria after a report of resistance, malaria treatment is not given as a single drug but in combination with artemisinin-based combination therapy (ACT) [6]. Efforts are needed to develop new antimalarial drugs from synthetic materials and natural materials that empirically have antimalarial activity, mainly plant-derived. One plant of the genus *Sauropus* was shown to have antimalarial activity against *P. falciparum* K1 strain from the 90% methanol fraction of *S. spatulifolius* leaves with an IC_50_ of 6.10 *µ*g/mL [7].

Based on genus and chemotaxonomic approaches, it is thought that the leaves of *S. androgynus* also have the content of compounds as antimalarials against *P. falciparum*. *S. androgynus* belongs to the family Euphorbiaceae known as star gooseberry which is a tropical shrub plant as a leafy vegetable and is a medicinal plant that has high antioxidant potential, can lose weight, and launch breastfeeding [8]. In addition, it can also be used for the treatment of several diseases such as antimicrobial, anti-inflammatory, antipyretic, antispasmodic, antiulcer, antiallergic, antioxidant, antitumor, anticancer, antiulcer, immunostimulant, and lipoxygenase inhibitor [9]. *S. androgynus* contains macronutrient and micronutrient components. It also contains polyphenols, vitamins, saponins, tannins, alkaloids, glycosides, and essential minerals including sodium (Na), potassium (K), phosphorus (P), iron (Fe), magnesium (Mg), copper (Cu), zinc (Zn), manganese (Mn), and cobalt (Co) [8]. Scientific information on biological activity, especially in relation to malaria, of *S. androgynus* leaves is still scarce. In vitro antiplasmodial activity of *S. androgynus* against the human malaria parasite *P. falciparum* was first reported by Mahardiani et al. [10]. In the preliminary study, n-hexane, chloroform, and 96% ethanol extract from *S. androgynus* leaves showed antiplasmodial activity against *P. falciparum* 3D7 strain with IC_50_ values of 1.23, 0.85, and 1.88 *μ*g/mL, respectively, and were shown to be nontoxic in human liver cancer cell lines. According to Berthi et al. [11], the antiplasmodial activity of the extract is considered to be very active with IC_50_ < 5 *μ*g/mL, promising with IC_50_ 6–15 *μ*g/mL, moderate with IC_50_ 16–30 *μ*g/mL, low with IC_50_ 31–50 *μ*g/mL, and inactive if IC_50_ > 50 *μ*g/mL. Thus, the antiplasmodial activity shown by n-hexane, chloroform, and ethanol extracts in the study was classified as very active (IC_50_ < 5 *μ*g/mL). Nevertheless, n-hexane and chloroform are among the categories of solvents that should be restricted in pharmaceutical products due to their toxicity properties [12]. Meanwhile, ethanol has been known as a good solvent for polyphenol extraction and is safe for human consumption [13]. Therefore, this study aims to identify the active fraction of 96% ethanol extract of *S. androgynus* leaves, and investigate the antimalarial activity of ethanolic extract using a mouse infection model as well as the inhibitory activity of the heme detoxification process in vitro.

## 2. Materials and Methods

### 2.1. Plant Sample Collection

*S. androgynus* leaves were obtained and determined from Purwodadi-LIPI Botanical Garden, Pasuruan, Indonesia with No. 074/68/102.7/2018.

### 2.2. Preparation of Fraction of Ethanolic Crude Extracts

The 96% ethanol extract of *S. androgynus* leaves produced by Mahardiani et al. [10] was used in this study and fractionated using VLC. The extract (2.505 g) was added with 85.015 g of silica gel 60 (0.040–0.063 mm) to dry and homogeneous, then put into a sintered glass containing two-thirds of full silica gel 60. The number of mobile phases used in each elution was 40 mL with a mixture of solvents of increased polarity, namely n-hexane, chloroform, and 96% ethanol. The mobile phase is poured into the sintered glass through filter paper and drawn off with a vacuum pump. The filtrate is housed in a vial for subsequent phytochemical screening using thin layer chromatography (TLC). Filtrates showing the same stains at phytochemical screening were combined and concentrated in a rotary evaporator at 45 rpm and 40°C, then concentrated to dryness with an oven at 36°C to obtain a dry fraction.

### 2.3. In Vitro Antimalarial Screening

*P. falciparum* 3D7 strain (chloroquine-sensitive) was used in this study to determine the in vitro antimalarial activity of *S. androgynus* leaf fraction. Parasites are routinely maintained according to the method described by Trager and Jensen [14] with slight modifications in the Laboratory of Pharmacognosy and Phytochemistry, Faculty of Pharmacy, Universitas Airlangga, Surabaya, Indonesia. *P. falciparum* cultures were maintained in human red blood cells (O+ blood type) with 5% hematocrit in Roswell Park Memorial Institute (RPMI) 1640 containing 2-(4-(2-hydroxyethyl)-1-piperazinyl)-ethanesulfonic acid (HEPES) 22.3 mM, hypoxanthine, sodium bicarbonate, gentamicin, and 10% human O+ plasma, and incubated at 37°C with 5% CO_2_.

The test was performed in duplicate in a 24-well well plate with 1% initial parasitemia. The *S. androgynus* leaf fraction was dissolved with dimethyl sulfoxide (DMSO) and prepared in various concentrations, and DMSO was used as the negative control. After 48 h of incubation, thin blood strips were prepared on glass slides, fixated in methanol, and stained with Giemsa. The percentage of parasitemia was calculated microscopically, then compared with a negative control for the determination of the percentage of inhibition of parasite growth. The IC_50_ value was calculated using probit analysis.

### 2.4. Experimental Animals and Parasite Inoculation

BALB/*c* mice, male, aged 6–8 weeks, and weighing 25–35 g, obtained from Pharmaceutical Veterinary Center (PUSVETMA), Surabaya, Indonesia, were used for the test. Mice were maintained in the veterinary laboratory of the Faculty of Pharmacy, Universitas Airlangga, Surabaya, Indonesia, under standard conditions at room temperature by exposing them to a cycle of 12 h of light and 12 h of dark, with food and ad libitum water. Mice are handled based on internationally accepted guidelines [15], and animal testing protocols have been approved by the Research Ethics Commission of the Faculty of Veterinary Medicine, Universitas Airlangga, Surabaya, Indonesia (No: 2.TO118.07.2018).

*P. berghei* ANKA strain was obtained from the Eijkman Institute for Molecular Biology, Jakarta, Indonesia and stored as frozen-infected erythrocytes stock in the parasitology laboratory of the Department of Parasitology, Faculty of Medicine, Universitas Airlangga, Surabaya, Indonesia. Parasites were maintained by inoculating *P. berghei*-infected erythrocytes in mice, and serial passage of blood from infected mice to uninfected mice was performed. Donor mice infected with increased parasitemia by 20–30% were used to infect test mice. Donor mice were dissected and intracardially blood was collected in microtubes containing 1% ethylenediaminetetraacetic acid (EDTA) as anticoagulant to avoid variability in parasitaemia. The blood was then diluted with phosphate-buffered saline (PBS) so that each 0.2 mL of blood contained 1 × 10^7^*P. berghei*-infected erythrocytes. Each mouse used in the experiment was then inoculated intraperitoneally with 0.2 mL of blood solution.

### 2.5. In Vivo Antimalarial Screening

#### 2.5.1. Evaluation of Suppressive Activity of Ethanolic Crude Extracts

In vivo antimalarial activity testing of ethanolic leaf extracts from *S. androgynus* was performed against *P. berghei* according to the method described by Peters et al. [16] by randomly assigning 36 male mice to 6 groups (4 treatment groups and 2 control groups). Four treatment groups received 1, 10, 100, and 200 mg/kg body weight of extract, respectively. Both controls (negative and positive) received 0.5% sodium carbocymethyl cellulose (Na CMC) and dihydroartemisinin-piperaquine (DHA-P, 20.8 + 166.4 mg/kg body weight), respectively. Na CMC, plant extracts, and DHA-P are administered once a day for 4 days orally. Treatment began after parasitemia in mice reached 1% (*D*_0_), using a hypodermic needle, and then continued for an additional 3 days (from *D*_1_ to *D*_3_). On day 5 (*D*_4_), thin blood strips were made from the tails of each mouse on a microscope slide. The blood was fixed with methanol, stained with 10% of Giemsa at pH 7.2 for 15 min and parasitemia was observed microscopically to determine the percent parasitaemia and the percent inhibition of parasites. Furthermore, data on the percent inhibition of parasites were used to calculate the ED_50_ value through probit analysis.

#### 2.5.2. Evaluation of Prophylactic Activity of Ethanolic Crude Extracts

**Figure 1:**
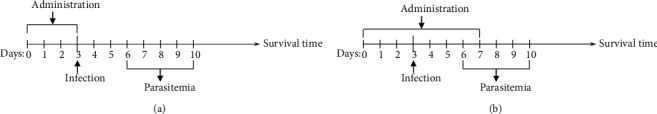
Test scheme of antimalarial activity prophylaxis in vivo of ethanolic leaf extract of *S. androgynus* against *P. berghei*. (a) Administration of extracts 4 consecutive days before parasitic infection (groups 1–4). (b) Administration of the extract 4 days before the parasitic infection and continued 4 days after the parasitic infection (group 5–8).

Prophylactic activity of ethanolic leaf extracts of *S. androgynus* and doxycycline was assessed using the method described by Peters [17] with modifications. Mice were randomly divided into eight groups, five each. Groups 1–3 were orally administered 100, 200, and 400 mg/kg body weight of extract, groups 4 and 5 were orally administered doxycycline (standard drug), and groups 6 and 7 were orally administered 100 and 200 mg/kg body weight of extract, respectively. Group 8 was given 0.5% Na CMC as a negative control. Administration of plant extracts or standard drugs in groups 1–4 was performed for 4 consecutive days (*D*_0_−*D*_3_). On day 4 (*D*_3_), mice were inoculated with *P. berghei* (see Figure 1(a)). While administration of standard drugs, plant extracts, or Na CMC in groups 5–8 was carried out for 4 consecutive days (*D*_0_−*D*_3_), on the fourth day (*D*_3_) inoculation with *P. berghei* was carried out, then followed by administration of extracts, standard drugs, or Na CMC for 4 consecutive days (*D*_4_−*D*_7_; see Figure 1(b)) [18]. The percent parasitemia of each mouse from the entire group was assessed by blood elimination 72–168 h after parasite inoculation (*D*_6_−*D*_10_).

Mortality was monitored daily and the number of days from parasite inoculation to death was recorded for each mouse in the trial and control group during the experimental period. The mean survival time (MST) for each group is calculated using the equation.

(1) $$\begin{array}{c} \text{MST}=\frac{\text{Total survival time of all mice in the group }\left(\text{day}\right)}{\text{Total number of mice in the group}}\text{.} \end{array}$$

### 2.6. Heme Detoxification Inhibitory Activity of Ethanolic Crude Extracts

The heme detoxification inhibitory activity was assessed using the Basilico method [19] with slight modifications. Each 10 mg of ethanolic leaf extract of *S. androgynus* and chloroquine diphosphate (positive control) were dissolved in 1 mL as a test stock solution (100 *µ*L DMSO and 900 *µ*L aquadest), then a solution of the test material was prepared with different concentrations at 4, 2, 1, 0.5, 0.25, and 0.1 mg/mL. About 100 *µ*L of a 1 mM hematin solution in 0.2 M NaOH was introduced into the microtubes, then 50 *µ*L of the test material solution was added. The heme detoxification reaction was initiated by adding 50 *µ*L of glacial acetic acid (pH 2.6) and incubating at 37°C for 24 h to allow optimum detoxification. The microtubes were then centrifuged at 8000 rpm for 10 min, the filtrate was separated, and the remaining pellets were resuspended with 200 *µ*L of DMSO. Washing with DMSO was performed three times. The pellet obtained is dissolved in 200 *µ*L of 0.1 M NaOH, herein after referred to as hematin alkaline solution. About 100 *µ*L of hematin alkaline solution was distributed into 96-well microplates, and the absorbance was read using an ELISA reader at a wavelength of 405 nm. The preparation of the standard curve for hematin starts from the preparation of a 1 mM hematin solution in NaOH 0.2 M which is then made up of the concentrations of hematin standard solutions 500, 250, 125, 62.5, 31.25, and 15.625 mM.

### 2.7. Statistical Analysis

The results of the study were expressed as mean ± standard error of mean (SEM). Data were analyzed using probit analysis. One-way analysis of variance (ANOVA) was used to determine statistical significance for the comparison of parasitemia and percent inhibition, among groups. The analysis was performed with a 95% confidence interval and a *p*-value of less than 0.05 was considered statistically significant.

## 3. Results

### 3.1. In Vitro Antimalarial Activity of Fractions of *S. androgynus*

The ethanolic leaf extract of *S. androgynus* was selected for activity-guided fractionation with VLC using a gradient of n-hexane-chloroform-ethanol 96% with a 10% concentration gradient. The fractionation results were then tested for in vitro antimalarial activity against *P. falciparum*. Based on the results of probit analysis, the IC_50_ value for fraction FV was 2.042 *µ*g/mL which is included in the active category as antimalarial. While fraction FII has an IC_50_ value of >10 *µ*g/mL and fractions FI, FIII, FIV, FVI, and FVII have an IC_50_ value of >50 *µ*g/mL, it is declared inactive antimalarial (see Table 1).

### 3.2. Effect on Suppressive Activity of Ethanolic Crude Extracts of *S. androgynus*

**Figure 2:**
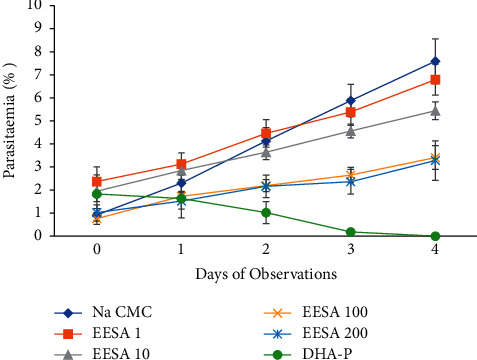
The effect of ethanolic leaf extract of *S. androgynus* on percent parasitemia of *P. berghei*-infected mice on 4-day suppression test. Data are expressed as mean ± SEM; *n* = 6; DHA-P = dihydroartemisinin-piperaquine, EESA = ethanolic extract; numbers refer to doses in mg/kg/day.

The extract shows a dose-dependent chemo-suppressive effect on parasitemia. This effect was statistically significant compared with the control (*p* < 0.05). The percent inhibition ranged from 33.73 to 66.11% (see Table 2). However, the effect of the extract was weak compared with the standard drug, DHA-P, with 100% inhibition (see Figure 2).

### 3.3. Effect on Prophylactic Activity of Ethanolic Crude Extracts of *S. androgynus*

The ethanolic leaf extract of *S. androgynus* showed prophylactic effects during prophylactic studies. This effect was statistically significantly different compared with the negative control (*p* < 0.05), but weak compared with the standard drug, doxycycline (see Table 3).

In addition, the percent inhibition between the administration groups 4 days before the infection (doses of 100 and 200 mg/kg body weight) resulted in a lower percentage of inhibition than the administration group continued after the infection (4 days before and after infection; doses of 100 and 200 mg/kg body weight), in the positive control group the administration before the infection and the administration continued after the infection resulted in a not too different percentage of inhibition which in the administration before the infection resulted in 69.75% inhibition (see Table 3) while in the administration group, continued after the infection resulted in 70.23% inhibition (see Table 4).

The group given the ethanolic leaf extract of *S. androgynus* survived longer than the negative control group (see Table 5).

### 3.4. Heme Detoxification Inhibitory Activity of Ethanolic Crude Extracts of *S. androgynus*

The ethanolic leaf extract of *S. androgynus* showed an effect in inhibiting the formation of *β*-hematin greater than 60% at a concentration of 2 mg/mL. The percentage of inhibition of *β*-hematin formation of ethanol extract and chloroquine diphosphate (positive control) shows that the higher the level of test material, the higher the percentage value of inhibition of *β*-hematin formation. The IC_50_ values of ethanolic leaf extract of *S. androgynus* and chloroquine diphosphate were 0.479 and 0.569 mg/mL, respectively (see Table 6). According to Baelmans et al. [20], compounds that have an IC_50_ value of inhibition of heme detoxification are smaller than the IC_50_ value of chloroquine sulfate, which is 12 mg/mL, so the compound can be categorized as having activity in inhibiting heme detoxification. So that the ethanolic leaf extract of *S. androgynus* can be said to have heme detoxification inhibitory activity.

## 4. Discussion

*Sauropus androgynus* (L.) Merr., based on a genus and chemotaxonomic approach with *S. spatulifolius* proven to have antimalarial activity, was investigated for in vitro and in vivo antimalarial activities using standard models. The results of in vitro research revealed that *S. androgynus* leaf extract has antimalarial activity against chloroquine-sensitive strain (3D7) of *P. falciparum* with an IC_50_ value of 96% ethanol extract of 1.88 *µ*g/mL which is classified as very active [10]. Therefore, fractionation was performed on 96% ethanol extract and in vitro antimalarial activity test on the results of fractionation against *P. falciparum*. The results showed that fraction FV of the seven fractions had the highest antimalarial activity (IC_50_ = 2.042 *µ*g/mL). However, the antimalarial activity of fraction FV was lower than that of 96% ethanol extract (IC_50_ = 1.88 *µ*g/mL). This result is in line with other findings by Ochieng et al. [21], where raw extracts from the aerial part of *Gardenia ternifolia* showed potent in vitro antimalarial activity against *P. falciparum* compared with its fractions and pure isolates. Crude extract of *G. ternifolia* roots also exhibited promising antimalarial activity against rodent malaria models, higher than its solvent fractions [22]. The reduction in antimalarial activity in the fraction compared with crude extract can be caused by the loss of synergistic activity between the compounds in the fraction [23]. Thus, 96% ethanol extract of *S. androgynus* leaves was selected for further evaluation in mice infected with *P. berghei* and its inhibitory activity against heme detoxification.

In vivo antimalarial activity can be classified as moderate, good, and very good if the extract displays, respectively, a percentage of parasite suppressors equal to or greater than 50% at doses of 500, 250, and 100 mg/kg body weight per day [24, 25]. Based on this classification, the ethanolic leaf extract of *S. androgynus* is considered to have shown good antimalarial activity, with dose-dependent inhibition against *P. berghei* infection in mice. In addition, comparisons between suppressive and prophylactic models have revealed that extract at 200 mg/kg body weight is more effective in inhibiting *P. berghei* growth in mice with inhibitions of 66.11 and 51.07%, respectively. Lower inhibitory effects in prophylactic tests may arise from rapid metabolism that inactivates the active components of the extract responsible for antimalarial activity [26]. This effect can also be seen in two different prophylactic models where at 200 mg/kg body weight of extract provides greater inhibition when administered for 8 days compared with 4 days of administration. This finding is consistent with other studies in which the inhibitory effect of prophylactic tests was lower than the 4-day suppressive test [27, 28]. However, the overall ethanolic leaf extract of *S. androgynus* significantly reduced parasitemia in prophylactic and suppressive models of *P. berghei*-infected mice confirming the antimalarial potential of this extract. These findings were further supported by the results of the mean survival time of mice given extracts that were significantly prolonged compared with those from the control group, indicating a significant protective potential of the extracts.

In testing the mechanisms and targets of antimalarial active compounds in killing parasites, several methods were carried out, one of which was by testing the inhibitory activity of heme detoxification into hemozoin by *P. falciparum* which was the focus of antimalarial drug research [19]. Some properties of hemozoin that are structurally similar to *β*-hematin make *β*-hematin an ideal product of the heme synthesis detoxification process [29]. The inhibitory effect of the heme detoxification process by ethanolic leaf extract of *S. androgynus* was studied in accordance with the results of the *β*-hematin barrier test in vitro. The test results revealed that the extract had the effect of inhibiting the formation of hemozoin which was characterized by the small value of *β*-hematin levels formed after incubating for 24 h (see Table 6). The inhibitory effect given was about 26–79% at a concentration of 0.1–4 mg/mL, and resulted in an IC_50_ value of 0.479 mg/mL. Frölich et al. [30] suggested that compounds with inhibition of hemozoin formation greater than 60% were declared to have good potential as hemozoin inhibitors. Thus, the ethanolic leaf extract of *S. androgynus* can be considered to have heme detoxification inhibitory activity. This inhibitory activity can result in death of parasites, which will minimize the pathogenesis of malaria, reduce the level of parasitemia, and reduce the accumulation of hemozoin in the liver and spleen of malaria patients which is one of the causes of hepatosplenomegaly.

Several secondary metabolites from plants such as alkaloids, flavonoids, and triterpenoids have been reported to have antimalarial activity [31–33]. Terpenoids have an important role as antimalarial agents by inhibiting the *Plasmodium* parasites' growth from ring forms to trophozoites, and can inhibit nutrient uptake by inhibiting the permeation pathway [34, 35]. Some antimalarial terpenoids isolated from other Euphorbiaceae plants have been reported, such as betulinic acid from *Uapaca nitida* Müll-Arg [36]; 8, 9-secokaurane diterpenes from *Croton kongensis* Gagnep [37]; geranylgeraniol from *Croton lobatus* L. [38]; poly-*O*-acylated jatrophane diterpenes from *Pedilanthus tithymaloides* (L.) Poit [39]; steenkrotin A from *Croton steenkampianus* Gerstner [40]; 2*α*-hydroxyjatropholone from *Jatropha integerrima* Jacq [41]; jatrophone diterpenes from *Jatropha isabelli* Müll. Arg [42]; 6-hydroxy neomacrolactone from *Neoboutonia macrocalyx* L. [43]; samvisterin from *Uapaca paludosa* [44]; euphorbesulin G from *Euphorbia esula* L. [45]; and many more. All these compounds exhibited good antimalarial activity with IC_50_ of ≤5 *μ*g/mL against various *P. falciparum* strains. This current study did not isolate the pure compound of S*. androgynous*, however, it was reported to contain various compounds. A number of 20 compounds were identified and the major compounds in the leaves extract that were detected in the present study were L-(+)-ascorbic acid 2, 6-dihexadecanoate (27.82%). They are followed by hexadecanoic acid, ethyl ester (17.85%); ethyl 9, 12, 15-octadecatrienoate (16.32%); ethyl (9Z, 12Z)-9, 12-octadecadienoate (9.40%); 9, 12, 15-octadecatrienoic acid, (Z, Z, Z)-(7.47%); 2, 6, 10-trimethyl, 14-ethylene-14-pentadecane (4.27%); phytol, acetate (4.12%); 9, 12-octadecadienoic acid (Z, Z)-(3.76%); 3, 7, 11, 15-tetramethyl-2-hexadecen-1-ol (2.04%); 2, 4-imidazolidinedione, 1-({(5-nitro-2-furanyl) methylene}amino)-(1.94%); octadecanoic acid, ethyl ester (1.39%); 7-octadecyne, 2-methyl-(0.94%); heptadecanoic acid, ethyl ester (0.59%); cyclopentasiloxane, decamethyl (0, 53%); cis-vaccenic acid (0.35%); 2, 6, 8-trimethyl-bicyclo [4.2.0]oct-2-ene-1, 8-diol (0.28%); 2-pentadecanone, 6, 10, 14-trimethyl- (0.27%); 9-octadecenoic acid (Z)- (0.26%); cyclohexasiloxane, dodecamethyl-(0.26%); and 1, 2-benzenedicarboxylic acid, 2-ethoxy-2-oxoethyl methyl ester (0.14%) [46]. Those compounds may the responsible for antimalarial activities. Mahardiani et al. [10] also have been reported that ethanolic leaf extract of *S. androgynus* was found in this study to contain terpenoids. The terpenoid content of *S. androgynus* leaves includes sesquiterpenoids and triterpenoids [9]. Thus, we suspect that the terpenoid content contained in this plant extract may have contributed to the antimalarial activity of this extract and therefore explains the mechanism of the antimalarial effect of the extract.

## 5. Conclusions

In general, the results of the current study indicate that 96% ethanol extracts and its fractions of *S. androgynus* leaves have potent antimalarial activity. The results also confirm that the plant has a suppressive and prophylactic effect on the growth of parasites. Ethanolic extract was also found to be active in inhibiting the heme detoxification process. This adds to the supporting evidence that the plant *S. androgynus* has the potential to be antimalarial from natural sources.

## Figures and Tables

**Table 1:** IC_50_ value of the fraction from ethanolic leaf extract of *S. androgynus*.

Fractions	Yields (%)	IC_50_ (*μ*g/mL)
FI (1, 2, 3)	0.12	5044.719
FII (4, 5)	0.12	17.401
FIII (6, 7, 8, 9)	0.72	575175.293
FIV (10, 11, 12, 13)	1.08	1908.618
FV (14, 15, 16)	39.24	2.042
FVI (17, 18, 19)	51.30	2576.625
FVII (20, 21)	11.94	988.354

**Table 2:** In vivo suppressive activity of ethanolic leaf extract of *S. androgynus* (4-day suppressive test) against *P. berghei*-infected mice.

Drug/Extract	Dose (mg/kg/day)	% Parasitaemia		% Suppression	ED_50_ (mg/kg bw)
		Day 0	Day 4		
Na CMC	—	0.92 ± 0.27	7.59 ± 0.97	—	—

Ethanolic leaf extract of *S. androgynus*	1	2.37 ± 0.64	6.79 ± 0.67	33.73	15.35
	10	1.95 ± 0.46	5.44 ± 0.38	47.67	
	100	0.76 ± 0.25	3.41 ± 0.51^*∗*^	60.26	
	200	1.02 ± 0.34	3.28 ± 0.86^*∗*^	66.11	

DHA-P	20.8 + 166.4	1.83 ± 0.83	0.00 ± 0.00^*∗*^	100.00	—

Data are expressed as mean ± SEM; *n* = 6; significance relative to the negative control, ^*∗*^*p* < 0.05.

**Table 3:** Percentages of inhibition of parasite growth in mice infected with *P. berghei* treated orally by doses of ethanolic leaf extract of *S. androgynus* for four consecutive days before infection (*D*_0_−*D*_3_).

Treatments	Dose (mg/kg/day)	% Parasitaemia		% Inhibition
		Day 6	Day 10	
Na CMC	—	0.26 ± 0.26	11.65 ± 1.50	—

Ethanolic leaf extract of *S. androgynus*	100	1.16 ± 0.39	6.61 ± 1.06	43.36 ± 18.38
	200	0.73 ± 0.27	5.70 ± 1.27	51.07 ± 21.77
	400	0.58 ± 0.38	3.76 ± 0.48^*∗*^	67.74 ± 9.21

Doxycycline	13	0.20 ± 0.12	3.52 ± 0.44^*∗*^	69.75 ± 8.51

Data are expressed as mean ± SEM; *n* = 5; significance relative to the negative control, ^*∗*^*p* < 0.05.

**Table 4:** Percentages of inhibition of parasite growth in mice infected with *P. berghei* treated orally by doses of ethanolic leaf extract of *S. androgynus* for 8 consecutive days before and after infection (*D*_0_−*D*_7_).

Treatments	Dose (mg/kg/day)	% Parasitaemia		% Inhibition
		Day 6	Day 10	
Na CMC	—	0.26 ± 0.26	11.65 ± 1.50	—

Ethanolic leaf extract of *S. androgynus*	100	1.00 ± 0.14	5.51 ± 0.13^*∗*^	52.70 ± 1.93
	200	0.92 ± 0.15	4.04 ± 0.54^*∗*^	65.30 ± 10.44

Doxycycline	13	0.00 ± 0.00	3.47 ± 0.36^*∗*^	70.23 ± 6.88

Data are expressed as mean ± SEM; *n* = 5; significance relative to the negative control, ^*∗*^*p* < 0.05.

**Table 5:** The mean survival time (MST) of mice receiving various doses of ethanolic leaf extract of *S. androgynus* during prophylactic study.

Treatments	Dose (mg/kg/day)	MST	
		Day 10	Day 17
Na CMC	—	0.8	0

Ethanolic leaf extract of *S. androgynus* for 4 consecutive days (*D*_0_-*D*_3_)	100	0.8	0.4
	200	0.8	0.4
	400	1	0.6

Doxycycline for 4 consecutive days (*D*_0_−*D*_3_)	13	1	0.6

Ethanolic leaf extract of *S. androgynus* for 8 consecutive days (*D*_0_−*D*_7_)	100	0.6	0.4
	200	1	0.8

Doxycycline for 8 consecutive days (*D*_0_−*D*_7_)	13	1	0.8

**Table 6:** The IC_50_ values of ethanolic leaf extract of *S. androgynus*, chloroquine diphosphate, negative control based on the inhibition test of heme detoxification.

Drug/Extract	Concentration (mg/mL)	Concentration of hemozoin (mM)	% Inhibition	IC_50_ (mg/mL)
DMSO	—	166.13 ± 11.48	—	—

Ethanolic leaf extract of *S. androgynus*	4	34.28 ± 1.90	79.36 ± 1.14	0.479
	2	50.29 ± 2.50	69.73 ± 1.50	
	1	67.59 ± 5.81	59.31 ± 3.50	
	0.5	81.57 ± 2.98	50.90 ± 1.80	
	0.25	95.31 ± 2.68	42.63 ± 1.62	
	0.1	121.48 ± 3.66	26.87 ± 2.20	

Chloroquine diphosphate	4	44.80 ± 3.10	73.04 ± 1.87	0.569
	2	56.42 ± 2.60	66.04 ± 1.56	
	1	69.82 ± 4.23	57.97 ± 2.55	
	0.5	84.69 ± 2.25	49.02 ± 1.35	
	0.25	102.66 ± 3.09	38.21 ± 1.86	
	0.1	118.17 ± 4.31	28.87 ± 2.60	

## Data Availability

The data used to support the findings of this study can be obtained from the corresponding author upon request.
